# Alterations in the rate of fertility, egg viability, and hatch parameters of adult geese exposed to different breeding methods

**DOI:** 10.1093/tas/txae094

**Published:** 2024-06-14

**Authors:** Elizabeth T Akinbola, Emmanuel O Ewuola

**Affiliations:** Department of Animal Science, Faculty of Agriculture, University of Ibadan, P.M.B. 5017, Ibadan, Oyo State, Nigeria; Department of Animal Science, Faculty of Agriculture, University of Ibadan, P.M.B. 5017, Ibadan, Oyo State, Nigeria

**Keywords:** breeding systems, egg viability, hatchability, artificial insemination, natural mating

## Abstract

In a 10-wk study, alterations in the rate of fertility, egg viability, and hatch parameters of adult geese exposed to different breeding methods were investigated. Twenty-four matured geese (4.0 ± 0.45 average weight) were randomly divided into three groups (TNM—natural mating group, TIM—artificial insemination group, TNI—natural mating and insemination group) of two replicates with four geese per replicate in a completely randomized design. Fresh semen collected from six ganders (5.2 ± 0.69 average weight) was pooled and used to inseminate the geese in TIM and TN1 at 0.2Ml at insemination times. The geese in TNM and TNI were allowed to mate naturally. Insemination and mating was done at 3 d interval and eggs from each treatment were collected daily. Incubation of eggs was done weekly, candling and transfer to hatcher were done on day 27 and goslings hatched out on day 30. Fertility, early embryo mortality (EEM), mid embryo mortality (MEM), late embryo mortality (LEM), hatch of fertile eggs (HOF), and hatch of set eggs (HOS) were obtained and analyzed using descriptive statistics and ANOVA and means separated using least significant difference test. Geese in TNI had significantly higher fertility (93.33 ± 10.97%) than TNM (59.67 ± 31.29%) and TIM (83.60 ± 17.14%). The EEM was higher in TIM than in the two other groups while the HOF and HOS were higher in TNM and TNI than in TIM. This study suggests that in comparison with TIM, higher fertility, hatchability, and lower embryo mortality can be obtained when geese are inseminated and naturally mated simultaneously.

## Teaser

The ultimate goal of investigating the most productive breeding method that can be adopted for geese is to immensely increase their productivity, as this species suffers from low egg fertility in Nigeria and many other countries. The application of artificial insemination in this research led to a huge increase in the percentage of fertility of geese eggs compared to the natural mating method. Moreover, the simultaneous introduction of natural mating with artificial insemination led to a higher increase in the egg fertility of the geese.

## Graphical Abstract

**Graphical Abstract:**
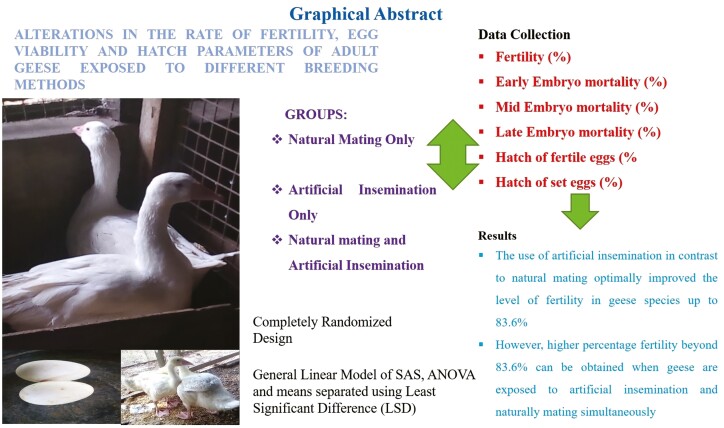

## Introduction

Indigenous geese meat is of high nutritive value meeting protein demand of humans while its production has good potential to increase farmers’ economic gain in terms of profit and national economy if produced on a commercial scale. However, the problem of low and uneconomic level of fertility in geese has hampered their commercialization over the years. Large-scale geese production is limited by their characteristic poor semen quality, low egg fertility, sexual behavioral demand for preferential mating or pairing and seasonal breeding (Łukaszewicz, 2010; Lukaszewicz et al., 2017). During natural mating, some associated problems include heavy body weight of the male compared with the female, which usually prevent proper cloaca contact, male leg disorder, and copulatory organ malformation and malfunction. These problems have often caused inefficient natural mating process (Łukaszewicz, 2010).

In addition, geese have a seasonal reproductive cycle, and the females lay fewer eggs. Hence, geese meat production is hampered throughout the year, despite the fact that the meat contains all the vital amino acids and suitable fatty acid profile (Heinz, 2011). Also, the volume of ejaculate produced by the males is always small with low live normal spermatozoa and spermatozoa concentration (Attila, 2020). These factors have led to the problem of low and uneconomic level of fertility in geese which has hampered their commercialization.

A favorable method reported in pedigree farms for productive use of best males, for a longer period of time is through artificial insemination. Some studies have been conducted in the temperate region to harness the use of artificial insemination in commercial geese production (Łukaszewicz, 2010; Brillard, 1993; Liu et al., 2008). In order to reduce the cost of producing gosling by decreasing the breeding male-to-female ratio needed on the farm and to improve fertility, artificial insemination may be required. Artificial insemination also limits the risks of transferring diseases associated with natural mating. Better productive use of ganders with high genetic potential to improve fecundity levels and accelerate genetic progress may also be possible by artificial insemination as compared to natural mating system (Blanco et al., 2009).

Thus, the mating difficulty in geese has necessitated the need to investigate the most productive breeding method for geese in order to improve the overall population of geese across Nigeria and globally. At present, little has been done to demonstrate the best breeding method that can be used for profitable commercial geese production in the tropical region of the world. Hence, the objective of this study was to investigate the alterations in the rate of fertility, egg viability, and hatch parameters of adult geese exposed to different breeding methods.

## Materials and Methods

Before the commencement of this study, all animal care, handling, and procedures were approved by the committee on research of the University of Ibadan, Ibadan, Nigeria—UI-ACUREC/19/0115.

### Experimental Site, Experimental Animals, and Management

The experimental location of the research was the Poultry Unit of the Teaching and Research Farm, University of Ibadan, Ibadan, Nigeria, Latitude 7° 26ʹ N and Longitude 3° 54ʹ E. Twenty-four (24) geese and six (6) ganders of 12 to 18 months old (average weight of 5.2 ± 0.7 kg for male and 3.9 ± 0.5 kg for female) were used for this study. Male and female geese were housed separately and were given feed and water ad libitum during the experimental period. Commercial layer’s mash (Brand name: Top Feed) containing crude protein of 16.5%, digestible energy of 2,500 kcal/kg, crude fiber of 6%, crude fat of 5%, calcium of 3.5%, and phosphorus of 0.41% was given to the birds throughout the experiment.

### Semen Collection and Evaluation

At the beginning of the experiment, semen was collected individually from all the ganders and evaluated microscopically. Pooled semen from 6 selected ganders was used for each insemination process. Also, a sample of the semen was evaluated at each insemination period for semen parameters such as semen volume, progressive spermatozoa motility, spermatozoa concentration and spermatozoa livability as described by Ewuola and Egbunike (2010). Using the dorsoabdominal massage method, semen was collected from the ganders individually (Johnson, 1954; Lukascewick, 2021). Before the commencement of semen collection, ganders were carefully captured and their wings were interlocked for movement restriction and curtailment. Then, they were massaged until their penile organ protruded out as described by Akinbola and Ewuola (2023). A collection tube was used to collect the dripping semen all through the penis canal length. Care was taken to prevent contamination of the semen with feces. Collected semen was pooled together and used for the insemination of the females. Through microscopic examination, semen volume, progressive spermatozoa motility, spermatozoa concentration, and ratio of live to dead sperm cells were evaluated (Abioja et al., 2022).

### Experimental Design and Insemination Procedures

Twenty-four geese were assigned to three treatment groups with two replicates with four geese per replicate in a completely randomized design. Geese in group 1 were mated naturally (TNM), geese in group 2 were artificially inseminated (TIM), and geese in group 3 were mated naturally and artificially inseminated (TNI). Insemination and mating were done at 3 d intervals for a period of 10 wk. The semen dosage for insemination was 0.2 mL as recommended by Akinbola and Ewuola (2023). Prior to insemination, each female goose was restrained by placing it on the lap and positioning its back section to face the first operator for ease of insemination while the head and neck faced the second operator. The Finger-guided method also known as the palpation method was used in the process of insemination of the females (Johnson, 1954; Łukaszewicz, 2002). This was done according to the modification of Akinbola and Ewuola (2023). In this method, the left index finger of the first operator was inserted into the vent of each bird in order to palpate the oviduct opening. A syringe with a glass tube attached, containing the semen was then inserted into the oviduct of the bird through the vent, and semen was deposited at a depth of about 3 to 4 cm (FAO, 2002; Łukaszewicz, 2002; Akinbola and Ewuola, 2023).

The geese in groups TNM and TNI were allowed to pair with the ganders of their choice before the onset of the experiment. Supervised natural mating was allowed at each mating period (3 d interval), in which the females were brought to the male pen and were taken out of the pen after a single successful mating. This was properly monitored for each female to prevent prejudice. The mating ratio used was 1:4 of males to females. Those artificially inseminated were exposed to insemination at 3 d interval also, while those naturally mated and inseminated received insemination and mating simultaneously at every 3 d.

### Egg Collection and Incubation

This experiment was carried out during the laying and breeding season of geese, between the month of December to February, of the year. Eggs from each group were collected daily, marked for identification before transfer to the hatchery. Incubation of eggs was done weekly for 10 wk. Candling was also done on day 27 to observe the growth and development of embryo in the fertile eggs and to separate infertile ones. The eggs were transferred to the hatcher on day 27 and goslings hatched out on day 30. Egg collection and incubation was done for 10 wk. The eggs candled out during the process of candling were broken to observe the state of development of their embryo before death. The stages of the embryonic development were recorded as either one of the following: early embryo mortality (days 1 to 10), mid embryo mortality (days 11 to 20) and late embryo mortality (days 21 to 30). Eggs noted to have no embryonic development were classified as infertile eggs according to the modification by Akinbola and Ewuola (2023).

### Data Collection

Data collected and recorded weekly include: egg fertility (%), hatch of fertile eggs (%), hatch of set eggs (%), early embryo mortality, mid embryo mortality (%), and late embryo mortality. Overall egg fertility was also determined after 10 wk


$$\begin{array}{l} egg\;fertility=\frac{number\;\;\;of\;\;\;fertile\;\;\;eggs}{number\;\;\;of\;\;\;set\;\;\;eggs}\;\;\;\times \;\;\;100 \end{array}$$

$$\begin{array}{l} hatch\;\;\;of\;\;\;fertile\;\;\;eggs\;(\;\%\;)=\frac{number\;\;\;of\;\;\;hatched\;\;\;goslings}{number\;\;\;of\;\;\;fertile\;\;\;eggs\;\;\;set}\times 100 \end{array}$$

$$\begin{array}{l} hatch\;\;\;of\;\;\;set\;\;\;eggs\;(\%)=\frac{number\;\;\;of\;\;\;hatched\;\;\;goslings}{number\;\;\;of\;\;\;set\;\;\;eggs}\;\;\;\;\;\times 100 \end{array}$$

$$\begin{array}{l} Embryo\;\;\;mortality\;(\;\%\;)=\frac{number\;\;\;of\;\;\;dead\;\;\;embryos}{number\;\;\;of\;\;\;set\;\;\;eggs}\;\;\;\;\;\times 100 \end{array}$$

### Statistics

Descriptive statistics (mean and range) was used to analyze the results of the individual and pooled semen of ganders to be used for insemination. One-way analysis of variance (ANOVA) at ^ἀ^_0.05_ using general linear model (GLM) of SAS (2003) was further used to analyze the overall egg fertility of geese exposed to natural mating only, artificial insemination only and natural mating coupled with artificial insemination. Through GLM, the relationship between the continuous and categorical predictors to the outcome variable was established. The weekly egg fertility result of geese in various groups is presented, with the aid of a bar chart. Differences in mean values were separated using Least Significant Difference (LSD) method due to identify the population whose means are statistically different. Beforehand, all data in percentages were subjected to log transformation (*x* + 1) before subjecting to one-way ANOVA. The results are presented as mean ± standard deviations to show the level of disparity of values in each group.

## Results

The semen quality parameter of ganders is shown in Table 1. The mean semen volume was 0.65 ± 0.23 mL while the spermatozoa motility ranged from 50.00% to 65.00% with a mean value of 55.83 ± 6.34%. The mean value of spermatozoa concentration was 9.09 ± 7.29 × 10^6^ sperm cells/mL and the mean live to dead ratio was 83.62 ± 14.47% with a range of 57.14% to 98.00% for all the ganders.

**Table 1.** Selected semen quality parameters of individual ganders

S/N	Semen volume, mL	Spermatozoa motility, %	Spermatozoa concentration (×10^6^ cells/mL)	Spermatozoa liveability, %
1	0.50	65.00	7.50	95.17
2	1.00	50.00	24.70	57.14
3	0.50	50.00	6.55	79.00
4	0.80	60.00	3.00	95.60
5	0.60	60.00	6.90	90.00
6	0.90	60.00	17.00	68.00
7	0.60	65.00	9.80	98.00
8	0.30	50.00	5.00	85.00
9	0.39	50.00	3.50	95.00
10	0.50	60.00	2.95	86.00
11	1.00	50.00	2.90	60.00
12	0.70	50.00	19.30	94.50
Mean ± SD	0.65 ± 0.23	55.83 ± 6.34	9.09 ± 7.29	83.62 ± 14.47
Range	0.30 to 1.00	50.00 to 65.00	2.90 to 24.70	57.14 to 98.00

The characteristics of pooled semen used for insemination at 0.2 mL over 10 wk duration is shown in Table 2. The mean progressive spermatozoa motility and spermatozoa livability were 64.00 ± 3.61% and 75.00 ± 4.58%, respectively, while the mean spermatozoa concentration was 19.50 ± 1.10** × **10^6^. Their ranges were 18.30 to 20.30 × 10^6^, 70% to 79% and 60% to 67%, respectively, for spermatozoa concentration, liveability, and progressive motility, respectively.

**Table 2.** Selected characteristics of pooled semen inseminated at 0.2 mL for 10 wk

Semen parameters	Mean values (± standard deviation)	Range
Spermatozoa concentration (×10^6^)	19.50 ± 1.10	18.30 to 20.30
Spermatozoa liveability, %	75.00 ± 4.58	70.00 to 79.00
Progressive spermatozoa motility (%)	64.00 ± 3.61	60.00 to 67.00

**Figure 1.**
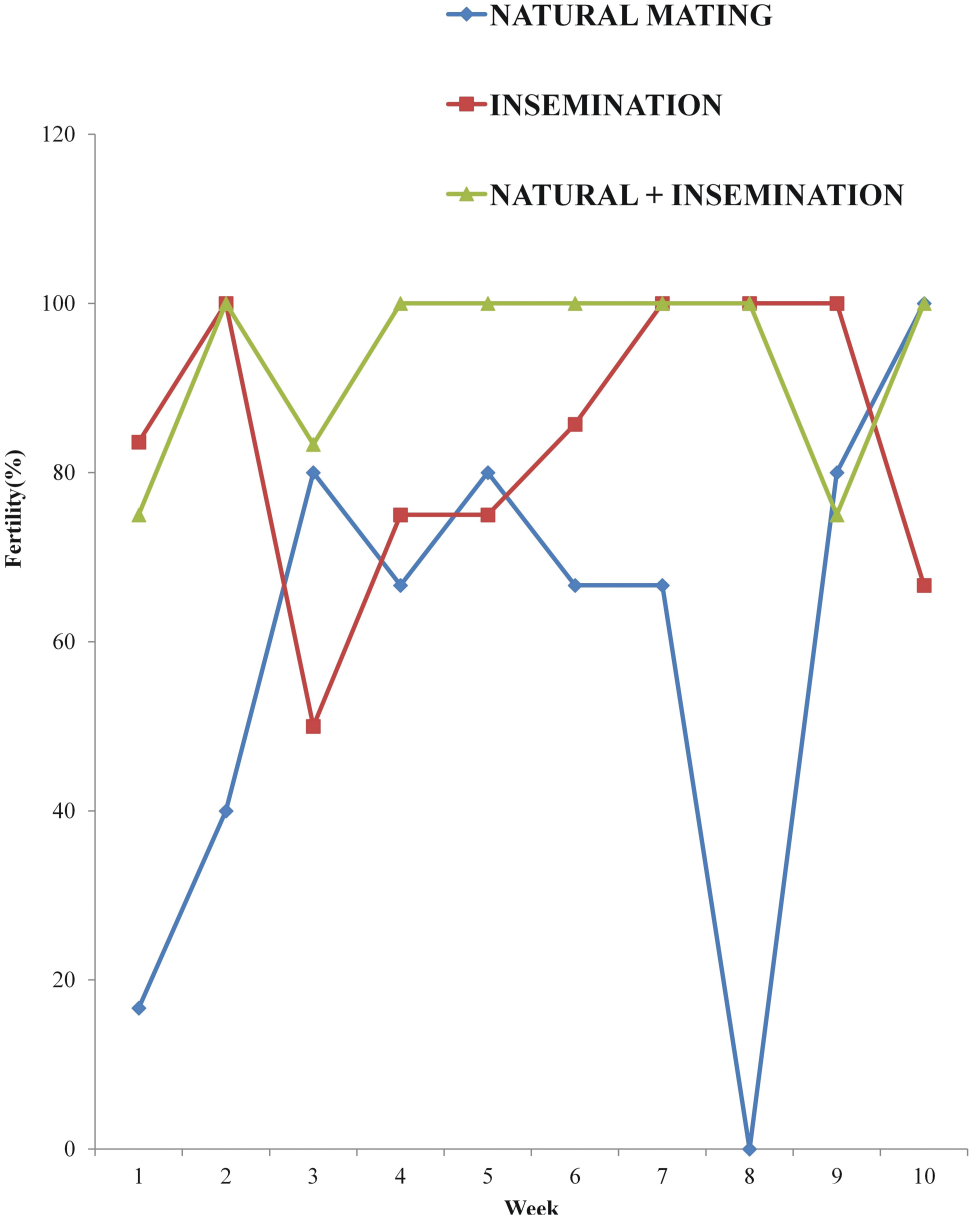
Weekly egg fertility pattern of geese mated and/or inseminated with undiluted semen for 10 weeks

The weekly trend of egg fertility for 10 wk is as shown in Figure 1. As shown in the figure, the geese exposed to natural mating had reduced egg fertility in all the 10 wk, as compared to those exposed to natural mating and insemination. Also, those inseminated only had higher fertility than those mated only, in most of the weeks (1, 2, 4, 6, 7, 8, and 9). The geese inseminated and naturally mated had higher egg fertility in weeks 4, 5, 6, and 10 as compared to those inseminated only. Table 3 shows the overall egg fertility of geese exposed to natural mating only, insemination only, and both insemination with natural mating. There were significant differences (*P* < 0.05) in groups. Geese exposed to insemination coupled with natural mating (TNI) had significantly (*P* < 0.05) higher fertility than those exposed to insemination only (TIM). Those exposed to insemination only (TIM) also had significantly (P < 0.05) higher fertility than those exposed to natural mating only (TNM). Their mean values were 59.67 ± 31.29%, 83.60 ± 17.14%, and 93.33 ± 10.97% for TNM, TIM, and TNI, respectively.

**Table 3.** Overall egg fertility of geese mated and/or inseminated with undiluted semen for 10 wk

Breeding methods	Fertility, %
Natural mating (TNM)	59.67 ± 31.29^c^
Insemination (TIM)	83.60 ± 17.14^b^
Natural mating + insemination (TNI)	93.33 ± 10.97^a^

^a,b^Mean in the same column with different superscripts are significantly different (*P* < 0.05).

The embryo mortality and hatch parameters of geese exposed to natural mating only, insemination only, and both insemination with natural mating are presented in Table 4. Significant differences (*P* < 0.05) were observed in the early embryo mortality values of the groups with the inseminated group having a higher value than the naturally mated group and the natural and inseminated group. The early embryo mortality (EEM) was higher in TIM than in the two other groups while the Hatch of Fertile eggs and Hatch of Set Eggs (HOF and HOS) were higher in TNM and TNI than in TIM. However, the Late Embryo Mortality was higher in TNM while Mid Embryo Mortality was not significantly different among groups.

**Table 4.** Embryo mortality and hatch parameters of geese mated and/or inseminated with undiluted semen for 10 wk

Breeding methods	EEM, %	MEM, %	LEM, %	Hatch of fertile eggs, %	Hatch of set eggs, %
Natural mating (TNM)	28.50 ± 12.25^c^	5.00 ± 0.00	34.50 ± 11.94^a^	22.00 ± 14.72^a^	17.00 ± 10.70^a^
Insemination (TIM)	86.67 ± 7.07^a^	5.56 ± 0.00	2.22 ± 0.00^c^	5.56 ± 0.00^b^	2.78 ± 0.00^b^
Natural mating + insemination (TNI)	48.12 ± 24.75^b^	12.83 ± 6.73	14.11 ± 10.64^b^	25.00 ± 12.95^a^	23.30 ± 15.53^a^

^a,b,c^Mean in the same column with different superscripts are significantly different (*P* < 0.05)

EEM, Early Embryo mortality; MEM, Mid Embryo Mortality; LEM, Late Embryo Mortality.

## Discussion

The individual gander semen quality showed lower spermatozoa motility and concentration compared to those of other male poultry species like cocks (male chicken) and toms (male turkey) (Adebisi and Ewuola, 2019; Ewuola et al., 2020). The values obtained were similar to those obtained for ganders according to some previous studies which reported that ganders produce mostly semen of low spermatozoa motility, spermatozoa concentration, and live normal spermatozoa that ranges between 50% and 60%, 71 and 337 × 10^6^ sperm cells/mL, and 14.1% and 34.7%, respectively (Łukaszewicz, 2002; Łukaszewicz et al., 2017; Akinbola, 2022; Akinbola and Ewuola, 2023; Ewuola et al., 2023). When compared with toms, cocks and drakes (male duck), poor status of semen is often observed in most breeds of ganders. Therefore, most qualitative and quantitative characteristics of their semen are low (Chełmońska, 1972). However, there were similarities in the number of live spermatozoa of ganders compared to other poultry species, (Łukaszewicz, 2002; Łukaszewicz et al., 2017). Rarely in few circumstances, the number of normal spermatozoa exceed 50% when gander semen is examined microscopically (Łukaszewicz, 2002; Liu et al., 2008).

The pooled semen quality of ganders that followed the same trend as the individual gander semen indicated that their semen had lower spermatozoa motility and concentration. The values obtained were similar to those obtained in ganders according to the studies of Akinbola and Ewuola (2023) and Łukaszewicz (2002) who also affirmed that ganders produce semen of low spermatozoa concentration and live normal spermatozoa. However, the number of live spermatozoa obtained from the pooled semen from the geese in this experiment (75%) was closer to the number obtained in other poultry species like toms (95.57%) and cocks (98.5%) (Ewuola et al., 2020; Abioye et al., 2022).

The lowest egg fertility value (59.67%) observed in the natural mating group is an indication for the difficulty of geese flock mating naturally. The success rate is lower in most cases. In geese farming systems where natural mating is used, problems such as excessive body weight, preferential mating, male leg disorder, and copulatory organ malformation cause retardation to their reproductive performance (Łukaszewicz, 2010, Lukaszewicz et al., 2017). Also in some cases, 80% of the ejaculated spermatozoa may regress from the vagina during natural mating (Bakst, 2011).

On the contrary, the fertility rate for the insemination group and the insemination with natural mating group (83.60% and 93.33%, respectively) were similar to 89% and 91.07% obtained for geese in the studies of Łukaszewicz (2002) and Akinbola and Ewuola (2023) for artificially inseminated geese. Also, the fertility result obtained for these group (83.60% and 93.30%) is comparable to the percentage fertility of artificially inseminated poultry species like turkey (99.27% to 99.84%) as reported by Olarinre et al. (2022). According to Łukaszewicz (2002), insemination of 9 million motile sperm cells weekly resulted to 89% fertility while in this study, 2.50 million motile sperm cells was inseminated every 3 d to obtain 83.60% fertility for TIM group and 93.33% for TNI group. Excellent fertility result above natural mating can be achieved through the use of only artificial insemination in geese. This may be due to several advantages that artificial insemination has over natural mating such as the elimination of preferential mating, prevention of disease transfers from males to females and removal of any male mating conformation defects such as copulatory organ malformation and malfunction (Penfold et al., 2000; Brillard, 2003; Fairchild and Christensen, 2005; Łukaszewicz, 2010). Also, during artificial insemination, there is direct introduction of sperm cells into the oviduct, as compared to the vagina deposition of semen during natural mating which may often allow for regression of semen from the vagina (Bakst, 2011).

However, the highest fertility value observed for geese in TNI group as compared with those in TIM and TNM groups is expected, and it gives an indication that combining artificial insemination with natural mating may be more may be more efficient in improving fertility and hatchability and also more profitable in commercial geese production. Although the use of artificial insemination singularly can also help to increase fertility beyond natural mating, combining insemination with natural mating may be more productive, since it employs the use of two breeding methods simultaneously, giving little chance for infertility.

Significantly higher early embryo mortality in the TIM group as compared to TNM and TNI groups could be due to the influence of uncontrollable hatchery managerial factors and conditions, on eggs set in this group since most of the eggs were fertile and were set randomly. Incubation traits in poultry may also be affected by several other factors such as fertilization rate, storage time, egg weight, genetic structure, feeding, age, and disease (Elibol and Türkoğlu, 2014; Kırmızıbayrak et al., 2016).

Moreso as reported, higher EEM is possible due to poor semen quality effect since the vagina was bypassed for introduction of semen directly into the oviduct, allowing large number of both physiologically normal and abnormal sperm cells to reach the infundibulum (Mclntyre et al.,1986; Bramwell and Howarth, 1997; Fairchild and Christensen, 2005). Lower late embryo mortality observed in TIM below what was obtained in TNI and TNM is expected since most of the embryo death recorded were early embryo mortality. Higher late embryo mortality in TNM is also expected since there was significantly lower early embryo death recorded.

As compared to TNM and TNI, lower rate of hatch of fertile eggs and hatch of set eggs in TIM could be possibly due to the high loss of most eggs in this group to embryo mortality especially early embryo mortality. In general, the low hatchability observed in all the breeding methods suggest that it is often common for the lowest egg hatchability to be found in geese eggs compared to eggs from many other poultry species when eggs are artificially incubated. The high embryo mortality and low hatchability observed in the study could be due to the longer incubation period of geese egg than other poultry species like layer chicken (21 d) and turkey (28 d). Geese incubation period varies from 30 to 35 d. This often leads to a lot of rotten eggs in incubated geese eggs. This same fact was pointed out in the report of Łukaszewicz et al. (2017) who indicated that for geese eggs to hatch, they require longer incubation periods than chicken, turkey, and many other poultry species. Thus, high embryo mortality and low number of hatched goslings are obtained yearly from the geese flock.

## Conclusions

The result of this study showed that the use of artificial insemination in contrast to natural mating optimally improved the level of fertility in geese species up to 83.6%, making it a reliable breeding practise needed to increase the number of goslings that can be produced yearly. However, compared to what was obtained in TIM, higher percentage fertility and hatchability with lower early embryo mortality can be achieved, when geese are exposed to artificial insemination and natural mating simultaneously. Significantly higher early embryo mortality in the TIM group compared to TNM and TNI groups could be due to the influence of hatchery managerial factors and other conditions of eggs set in this group. Also, poor semen quality effect could have led to fertilisation by both normal and abnormal sperm cells.

## References

[CIT0001] Abioja, M. O., S. Apuu, J. M. Daramola, M. Wheto, and O. F. Akinjute. 2022. Semen quality and sperm characteristics in broiler breeder cockerels fed vitamin E during hot season. Acta Scientiarum. Anim. Sci. 45(1). ISSN 1807-8672. doi:10.4025/actascianimsci.v45i1.56848

[CIT0002] Abioye, T. V., O.O. Adagba, S. A. Fabule, D. O. Adeyemi, I. O. Olarinre, E. T. Akinbola, and E. O. Ewuola. 2022. Reproductive assessment of indigenous turkeys at puberty based on their plumage colour. Proceedings of the 11th ASAN-NIAS Joint Annual Meeting held at Zaranda Hall, Bauchi, Bauchi State, October 23rd – 27th, 2022; 149–152.

[CIT0003] Adebisi, K. A., and E. O. Ewuola. 2019. Fertility response of indigenous turkey hens to semen dosage and oviductal spermatozoa storage. J. Vet. Andr. 4:33–39.

[CIT0004] Akinbola, E. T. 2022. Thermoregulatory assessment and egg fertility response of geese to artificial insemination (PhD thesis, University of Ibadan, Nigeria).

[CIT0005] Akinbola, E. T., and E. O. Ewuola. 2023. Effects of semen dose on egg fertility, embryo mortality and hatchability in artificially inseminated geese (*Anser cygnoides*). Bulgarian Journal of Veterinary Medicine. (online first) ISSN 1311–1477. doi:10.15547/bjvm.2023-0005

[CIT0006] Attila, S. 2020. Fertility and hatchability in goose eggs: A review. Int. J. Poult. Sci. 19:51–65. doi:10.3923/ijps.2020.51.65

[CIT0007] Bakst, M. R. 2011. Role of the oviduct in maintaining sustained fertility in hens. J. Anim. Sci. 89:1323–1329. doi:10.2527/jas.2010-3663 21169513

[CIT0008] Blanco, J. M., D. E. Wildt, U. W. Hofle, W. Voelker, and A. M. Donoghue. 2009. Implementing artificial insemination as an effective tool for ex situ conservation of endangeredavianspecies. Theriogenology. 71:200–213. doi:10.1016/j.theriogenology.2008.09.019 19004491

[CIT0009] Bramwell, R. K., and B. Howarth. 1997. Effect of low or high sperm penetration values at the germinal disc on early embryonic mortality in chicken eggs. Poult. Sci. 76:97.

[CIT0010] Brillard, J. P. 1993. Sperm storage and transport following natural mating and artificial insemination. Poult. Sci. 72:923–928. doi:10.3382/ps.0720923 8502613

[CIT0011] Brillard, J. P. 2003. Practical aspects of fertility in poultry. J. World's Poult. Sci. 59:441–446. doi:10.1079/wps20030027

[CIT0012] Chełmońska, B. 1972. Seasonal changes in ganders’ reproductive organ in artificial insemination aspect. Part II. Polskie Archiwum Weterynaryjne 15:575–96. PMID:4662593. 4662593

[CIT0013] Elibol, O. and M. Türkoğlu. 2014. Embriyo Gelişimi ve Kuluçka. In: Türkoğlu M, Sarıca M, editors: Tavukçuluk Bilimi (Yetiştirme, Besleme, Hastalıklar). 4th ed. Ankara: Bey Ofset Matbaacılık, 166–206.

[CIT0014] Ewuola, E. O., E. T. Akinbola, J. Oyewale, and A. Ogundele. 2023. Assessment of the reproductive performance of wallowed and non-wallowed geese at high temperature humidity index during breeding season. Zhivotnovadni Nauki. 60:21–29.

[CIT0016] Ewuola, E. O., and G. N. Egbunike. 2010. Gonadal and extra – gonadal sperm reserves and sperm production of pubertal rabbits fed dietary fumonism B_1_. Biological Journal of Animal Reproduction Science. 119:282–286. doi:10.1016/j.anireprosci.2009.12.001 20079583

[CIT0015] Ewuola, E. O., K. T. Ogundeji, T. M. Osanyinlusi, D. M. Oyedele, K. A. Adebisi, and O. A. Bolarinwa. 2020. Effects of semen dosage, oviductal sperm storage and insemination interval on egg fertility, embryo mortality and hatchability in Nera black breeder chickens. Nigerian Journal of Animal Production. 47:34–43. doi:10.51791/njap.v47i3.134

[CIT0017] Fairchild, B. D., and V. L. Christensen. 2005. Influence of hen age and number of inseminated sperm on the number of holes hydrolyzed in the inner perivitelline layer of Turkey Eggs. J. Appl. Poult. Res. 14:576–581. doi:10.1093/japr/14.3.576

[CIT0018] FAO, 2002. Goose Production Systems - Part 1. Italy: FAO Animal Production and Health Paper-Rome; 154.

[CIT0019] Heinz, P. L. 2011. Water Fowl Production for food Security. Lohmann Information. 46:40.

[CIT0020] Johnson, A. S. 1954. Artificial insemination and duration of fertility of geese. Poult. Sci. 33:638–640. doi:10.3382/ps.0330638

[CIT0021] Kırmızıbayrak, T., B. Boğa Kuru, and K. Yazıcı. 2016. Yield and traits of geese eggs and hatchability traits. Turkiye Klinikleri J Reprod Artif Insemin. 2:42–47.

[CIT0022] Liu, S. J., J. X. Zheng, and N. Yang. 2008. Semen quality factor as an indicator of fertilizing ability for geese. Poult. Sci. 87:155–159. doi:10.3382/ps.2007-00300 18079465

[CIT0023] Łukaszewicz, E. T. 2002. Cryoconservation of gander semen. Zesztyty Naukowe Akademii Rolniczej weWrocławiu, Poland 440:1–111.

[CIT0024] Łukaszewicz, E. T. 2010. Artificial insemination in geese. Proceeding In: World’s Poultry Science Journal. Wroclaw, Poland: Vol. 66. doi:10.1017/S0043933910000632

[CIT0026] Łukaszewicz, E. T. 2021. Characteristics of semen collected from gander included in the genetic resources conservation program. Poult. Sci. 100:101314. doi:10.1016/j.psj.2021.101314. PMID: 34352409 34352409 PMC8350414

[CIT0025] Łukaszewicz, E. T., M. Lason, J. Rosenberger, A. Kowalczyk, and M. Bakst. 2017. Goose embryonic development from oviposition through 16 hours of incubation. Journal of Poultry Science. 1:1934–1938. doi:10.3382/ps/pew474. PMID:28053196 28053196

[CIT0027] McIntyre, D. R., V. L. Christensen, and L. G. Bagley. 1986. Effect of sperm numbers per insemination following early or late initial inseminations in turkeys. Poult. Sci. 65:1400–1404. doi:10.3382/ps.0651400 3748951

[CIT0028] Olarinre, I. O., O. O. Adagba, T. V. Abioye, D. O. Adeyemi, S. A. Fabule, E. T. Akinbola, and E. O. Ewuola. 2022. Poult plumage colour characterisation of F1 progeny of crosses between white and black indigenous turkeys artificially inseminated. In: 11th ASAN-NIAS Joint Annual Meeting held at Zaranda Hall, Bauchi, Bauchi State, October 23rd – 27th, 2022; 153–156.

[CIT0029] Penfold, L. M., D. E. Wildt, T. L. Herzog, W. Lynch, L. Ware, S. E. Derrickson, and S. L. Monfort. 2000. Seasonal patterns of LH, testosterone and semen quality in the Northern Pintail duck. Reproduction, Fertility Development 12: 229–235. doi: 10.1071/rd00093 11302434

[CIT0030] SAS. 2003. Statistical analysis system. SAS Release 9.1 for Windows. Cary, NC: SAS Institute Inc.

